# Latent Autoimmune Diabetes in Adults (LADA): From Immunopathogenesis to Immunotherapy

**DOI:** 10.3389/fendo.2022.917169

**Published:** 2022-07-21

**Authors:** Jingyi Hu, Rong Zhang, Hailan Zou, Lingxiang Xie, Zhiguang Zhou, Yang Xiao

**Affiliations:** National Clinical Research Center for Metabolic Diseases, Key Laboratory of Diabetes Immunology, Ministry of Education, Department of Metabolism and Endocrinology, The Second Xiangya Hospital of Central South University, Changsha, China

**Keywords:** latent autoimmune diabetes in adults, immunopathogenesis, adaptive immunity, innate immunity, gut-associated immunity, immunotherapy

## Abstract

Latent autoimmune diabetes in adults (LADA) is a type of diabetes characterized by slow autoimmune damage of pancreatic β cells without insulin treatment in the early clinical stage. There are differences between LADA and classical type 1 diabetes (T1D) and type 2 diabetes (T2D) in genetic background, autoimmune response, rate of islet function decline, clinical metabolic characteristics, and so on. The disease progression and drug response of patients with LADA are closely related to the level of islet autoimmunity, thus exploring the pathogenesis of LADA is of great significance for its prevention and treatment. Previous studies reported that adaptive immunity and innate immunity play a critical role in the etiology of LADA. Recent studies have shown that the intestinal microbiota which impacts host immunity hugely, participates in the pathogenesis of LADA. In addition, the progression of autoimmune pancreatic β cell destruction in LADA is slower than in classical T1D, providing a wider window of opportunities for intervention. Therefore, therapies including antidiabetic drugs with immune-regulation effects and immunomodulators could contribute to promising interventions for LADA. We also shed light on potential interventions targeting the gut microbiota and gut-associated immunity, which may be envisaged to halt or delay the process of autoimmunity in LADA.

## 1 Introduction

Latent autoimmune diabetes in adults (LADA), a disease with a phenotype similar to type 2 diabetes (T2D), but with slow destruction of pancreatic β cells, has been recognized by the American Diabetes Association as a form of type 1 diabetes (T1D) in the 2022 classification (1–4). LADA accounts for approximately 2%-12% of all diabetic patients (2, 5), affecting more than 10 million individuals in China (6). Multicenter studies reported that 4% to 14% of patients initially diagnosed with T2D are further diagnosed as LADA based on autoantibody tests (7–9). As an autoimmune diabetes, LADA patients exhibit a mild autoimmune process, and β cell function declines more slowly (10). After onset, it tends not to require insulin therapy for at least more than 6 months in LADA patients (10). Therefore, there is a relatively long period before the patients develop pancreatic β cell failure. It is not only conducive to studying the mechanism of autoimmune destruction of β cells, but also provides a valuable time window for actively seeking new methods to prevent or delay the failure of β cells in LADA patients.

The pathogenesis of LADA has not yet been clarified, and many studies have shown that LADA is mainly caused by cell-mediated immunity (11, 12). *In vitro*, studies have found that peripheral blood mononuclear cells (PBMC) of patients with LADA can inhibit the secretion of insulin by human islets, suggesting that there is a cell-mediated autoimmune response in LADA (11). Japanese scientists performed a pancreatic biopsy on patients with LADA and found that the changes in insulitis were dominated by T cell infiltration, which is the most direct evidence of the onset of LADA mediated by cellular immunity (13). A reduced number of regulatory T cells and their functional defects were considered to be indispensable causes of autoimmunity in LADA (14). In recent years, more and more studies have shown that innate immunity is closely related to the pathogenesis of autoimmune diabetes (15–17). LADA and T1D may share some immunological features due to the presence of common identifiable pancreatic β cell-specific autoantibodies, similar cellular and systemic proinflammatory cytokine profiles, and consistent alterations in the immunophenotype of certain immune cells (18–20).

Current research focuses on the interaction between the intestinal microbiota and the immune system. Intestinal microbiota and metabolites can regulate the physiological and pathological conditions of the host through immune regulation. And for newborn mammals, the existence of symbiotic microbiota is necessary for the development of their immune system. Furthermore, the disturbance of the intestinal microbiota may also lead to impaired intestinal barrier function and leakage of toxic metabolites into the circulation. Substantial evidence supports the involvement of gut microbiota in the pathogenesis of multiple systemic autoimmune diseases (21). A recent study has shown that the gut microbiome and metabolite profiles of LADA patients are significantly different from healthy subjects and typical T1D and T2D patients, and the gut microbial structure of LADA patients is more similar to glutamic acid decarboxylase antibody (GADA)-positive T1D patients (22). Thus, revealing the pathogenic role of microbiota and related metabolites and targeting microbiota-immune axis may open up new ideas for the treatment of LADA. Here, we review the current research progress in autoimmune diabetes and immunology, mainly involving the role of adaptive immunity, innate immunity, and gut-associated immunity in the pathogenesis of LADA, and the potential immunomodulatory treatments.

## 2 Immunological Mechanisms of LADA

### 2.1 Adaptive Immunity and LADA

Adaptive immune cells such as T cells and B cells play important roles in autoimmune diabetes. Multiple studies have reported phenotypic alterations in T and B cells in LADA patients (23–27). Although previous studies have mainly described the phenomenon of cellular and humoral immunity in LADA patients, further mechanisms have recently been explored. The first genome-wide association study of LADA suggested that cytotoxic T cell-related signaling pathways were abnormal in LADA patients, supporting an important role of adaptive immunity in the pathogenesis of LADA (28).

#### 2.1.1 Adaptive Immune Cells and LADA

##### 2.1.1.1 T Cells

Autoreactive T cells are the main effector cells of β cell autoimmunity. In general, pancreatic β cells are damaged or die in response to various genetic and environmental factors to release autoantigens, and pancreatic draining lymph nodes undergo naïve T cell activation after encountering islet autoantigens, after which T cells migrate to infiltrate the islets (29, 30). Pancreatic biopsy results revealed that CD8^+^ T cells were one of the major contributors to immune cell infiltration in insulitis in patients with LADA (31). It is well-established that after recognizing antigenic determinants expressed on the surface of pancreatic β cells related to MHC-I molecules, autoreactive CD8^+^ T cells exert their killing effect on β cells by cytotoxic degranulation and release of perforin that helps synergistically release granzymes with serine protease activity into the cell (32). As mentioned before, although there was no significant difference in the total number of islet-infiltrating immune cells between T1D and LADA patients and their corresponding rat models, there were differences in immune cell composition, that is, lower level of CD8^+^ T cells in LADA compared with T1D (33). Perhaps, the above phenomenon is involved in the reason why islets in LADA are more mildly destroyed than those in T1D. In fact, Sachdeva et al. have found that compared with the T1D group, the frequency of peripheral islet antigen-specific autoreactive CD8^+^ T cells in the LADA group is lower and the central-memory subset is relatively restrained under *in vitro* stimulation with pancreatic β cell-associated antigen, and thus the autoreactive CD8^+^ T cells in LADA had inferior proliferative capacity, but their function was comparable between the two groups (24). These findings support an important role for CD8^+^ T cells in the pathogenesis of LADA, underscoring the importance of developing CD8^+^ T cells as therapeutic targets.

Infiltration of CD4^+^ T cells was also observed in the pancreas of LADA patients and rat models (33). Previous studies have reported that in autoimmune diabetes, in addition to contributing to pancreatic β cell death through secretion of cytokines (e.g., IFN-γ and TNF-α) and direct contact, CD4^+^ T cells stimulate macrophages for M1-like polarization, promote dendritic cells (DCs) to effectively stimulate CD8^+^ T cell responses, and contribute to the activation of B cells (34–39). However, more research has focused on the role of regulatory T cells (Tregs) in LADA patients. Tregs are important components of the immune system, which suppress proliferation and cytokine secretion of CD4^+^ T cells and CD8^+^ T cells, and reduce costimulatory ligand expression on APCs (40). Some clinical studies have reported significant reductions in the frequency and number of Tregs in LADA patients (41, 42). Other studies have found that the expression of FOXP3 (a Treg marker) mRNA in CD4^+^ T cells of LADA patients is significantly reduced than that of controls, and the FOXP3 promoter region is hypermethylated (14). IL-35, a novel cytokine, has been implicated in the maintenance of the normal suppressive phenotype of Tregs, and systemic administration of IL-35 has been shown to be effective in preventing the development of diabetes in the multiple low-dose streptozotocin (MLDSTZ) mouse model and reversing hyperglycemia in diabetic NOD mice (43). It has recently been shown that LADA patients have reduced levels of both IL-35^+^ Tregs and plasma IL-35 (42). These suggest that adoptive transfer of Tregs or reversing the abnormal DNA methylation or cytokine production pattern in Tregs may provide a new perspective for cell-specific treatment of LADA.

Cytotoxic T lymphocyte antigen-4 (CTLA-4) is an important negative regulator of T cell activation and expansion constitutively expressed on the surface of Tregs to mediate the suppressive functions of Tregs, and can also be detectable on the surface of activated conventional T cells (44–46). CTLA-4 functions at the cell surface but is primarily localized in intracellular vesicles in the Trans-Golgi network and secreted to the cell surface upon TCR activation (47, 48). Studies have shown that CTLA-4 gene polymorphism is associated with genetic susceptibility to LADA, and the distribution of CTLA-4 +49A/G genotype in LADA patients is associated with GADA titers (49, 50). Specifically, the G allele of the +49A/G SNP is associated with reduced control of T cell proliferation, which may aid in understanding the pathogenesis of LADA (51, 52). However, there are few studies on the role of T cell inhibitory receptors in LADA. Future investigations of T cell inhibitory receptors such as CTLA-4 are expected to verify the profound mechanisms and therapeutic potential in LADA.

##### 2.1.1.2 B Cells

Growing evidence also support a role of B cells in autoimmune-mediated β cell destruction. B cells produce islet autoantibodies to help identify the risk of autoimmune diabetes, and capture and present autoantigens to activate autoreactive CD4^+^ T cells and facilitate the survival and differentiation of CD8^+^ T cells (53–55). A previous study has shown no significant difference in the frequency of CD19^+^ B cells in peripheral blood of T1D and LADA patients compared with healthy subjects with normal glucose tolerance (NGT), but their B cell subsets are altered; it can be found that the percentage of marginal zone B (MZB) cells negatively correlated with fasting C-peptide (FCP) is increased and the percentage of follicular B (FOB) cells positively correlated with FCP is decreased (26). MZB cells are reported to activate naive CD4+ T cells more efficiently than FOB cells (56). In addition, it is noteworthy that the frequency of regulatory B cells (Bregs), which can modulate T cell responses and suppress inflammation by secreting cytokines such as IL-10, IL-35, etc., is lower in LADA patients than in T1D patients (26, 42). This further explains the immune protection mechanism of pancreatic β cell destruction more slowly in LADA. Clearly, an imbalance of pathogenic and regulatory B cells leads to a loss of immune homeostasis, and a better understanding of the interaction between T cells and B cells will also provide new insights into the pathogenesis of LADA.

#### 2.1.2 Islet Autoantibodies and LADA

The islet autoantibodies of LADA mainly include GADA, protein tyrosine phosphatase IA-2 autoantibody (IA-2A), zinc transporter 8 autoantibody (ZnT8A) and insulin autoantibody (IAA) (5). The presence and levels of islet autoantibodies are associated with phenotypical features and insulin requirements in LADA patients. Compared with T1D patients, LADA patients were more likely to have GADA and a higher frequency of N-terminal reactive GADA (57, 58). A prospective study showed that 56.1% of LADA patients progressed to require insulin therapy during a 7-year follow-up period, compared with 20.9% of T2D patients, and that high GADA titer increased the risk of insulin requirement in LADA patients (59). As an IA-2 fragment lacking the COOH-terminal portion of the protein, IA-2 (256–760) increases with increasing body mass index (BMI) in obese LADA patients (60). Low-grade inflammation associated with β cell damage could also determine adaptive autoimmunity against pancreatic β cells. Tiberti et al. have found that IA-2**_(256–760)_** fragment was the most sensitive marker for detecting humoral IA-2 immunoreactivity in LADA patients, which contained a number of IA-2 T cell epitopes recognized by human CD4**^+^** T cells (61). However, studies have shown that LADA patients only with IA-2_(256–760)_ antibody have milder autoimmune responses than LADA patients with high GADA titers, and progress to insulin treatment phase slowly, suggesting that this humoral autoimmune response, mainly represented by IA-2_(256–760)_ autoantibodies, may not necessarily play a pathogenic role (60, 62). Therefore, it is necessary to focus on the link between the autoantibodies and the phenotype in LADA patients to facilitate the understanding of the wide heterogeneity of LADA, or to predict β cell failure and guide individualized therapy.

### 2.2 Innate Immunity and LADA

In recent years, studies on the role of innate immune cells in LADA have gradually increased. The immune cells are mainly involved in innate immune responses including neutrophils, NK cells, macrophages, basophils, and eosinophils (63, 64). Innate immune cells kill infected microorganisms by production of inflammatory cytokines and chemokines, and phagocytosis. Innate immunity is an important step in triggering adaptive immunity. The changes in the frequency of macrophages, neutrophils, NK cells, and other innate immune cells indicate that they may be involved in β cell autoimmunity. Animal experiments have shown that pattern recognition receptors (PRRs) such as Toll-like receptor 2 (TLR2) can activate APCs such as DCs and macrophages to activate autoreactive T cells inducing β cell autoimmunity.

#### 2.2.1 Innate Immune Cells and LADA

##### 2.2.1.1 Macrophages

Macrophages are cellular components of the innate immune system and exist in almost all tissues. They are not only conducive to the internal environment homeostasis and repair, but also the main regulator of the immune response (65). Under different microenvironments, the function of macrophages shows heterogeneity (66). Macrophages are usually divided into two types, M1 type related to host defense and pro-inflammatory response, or M2 type polarization related to tissue repair and anti-inflammatory response (66).

In the status of hyperglycemia, the differentiation of macrophages is affected, resulting in increased expression of TLR2 and TLR4 and increased expression of inflammatory factors (67). Notably, the macrophages infiltration of the pancreas in patients with LADA was significantly more severe than that in patients with T2D and healthy individuals (33). Previous studies have found that pancreatic β cells are particularly sensitive to the cytotoxicity of macrophages (68). Islet macrophages can damage β function through cell-to-cell contact (69). When islet autoimmunity is initiated, islet macrophages may contribute to the development of autoimmune diabetes by presenting islet autoantigens to T cells (70). There is also evidence that in the pancreas of LADA patients and a first spontaneous rat model of LADA, immune cell infiltration transfers from CD8^+^ T cells to CD68^+^ macrophages, and the gene expression of proinflammatory factors transfer from TNF-α to IL-1β (33). Compared with other immune cells, macrophages produce more IL-1β (71), which is dominant in LADA, versus more TNF-α, which is dominant in T1D. The β cell cytotoxicity of TNF-α is higher than that of IL-1β (72).This may contribute to the slower progression of LADA compared to T1D (33). In addition, recent studies have found macrophages can create a lineage-specific microenvironment for the regeneration of mouse pancreatic β cells (73). Brissova M et al. have found that the proliferation of β cells depends on the recruitment of macrophages, and the cytokines produced by the recruitment of macrophages are beneficial to the proliferation of β cells (74). Pancreatic islet macrophages can sense β cell activation to promote the stability of islet composition (75). Whether macrophages become effector cells for the development of autoimmune diabetes or play a beneficial role in β cell proliferation and development depends on the stimulation of macrophages. Further research is needed to clarify the mechanism of macrophage heterogeneity and to explore whether it is possible to induce macrophage polarization to provide new therapies for autoimmune diabetes.

##### 2.2.1.2 Neutrophils

Neutrophils are one of the first innate immune cells recruited to inflammation sites to initiate antibacterial effects, including degranulation, phagocytosis, and the production of neutrophil extracellular traps (NETs) (76). More and more evidence indicate that neutrophils are involved in the occurrence and development of LADA. Our clinical studies have shown that the neutrophil counts of LADA patients are higher than those of T1D patients and lower than those of T2D patients (77). Neutrophil counts and number and titers of islet autoantibodies are closely related. Pancreatic macrophages and β cells can recruit neutrophils from the circulation to pancreatic islets (77). Meanwhile, we also found that neutrophils from LADA patients showed activation of various biological pathways such as degranulation, adhesion and migration at the transcriptional level compared with healthy subjects (78). Blocking the activity of neutrophils can reduce the development of insulitis and diabetes (79), suggesting that neutrophils play an important role in the early pathological process of autoimmune diabetes.

Neutrophil serine proteases, including neutrophil elastase (NE), protease 3 (PR3), and cathepsin G (CG), are the main components involved in the removal of neutrophil azurophilic granules that are engulfed by microorganisms (80). Among them, PR3, as one of the main target antigens of anti-neutrophil cytoplasmic antibody (ANCA), plays a key role in inflammation. Recent studies have shown that PR3 may mediate neutrophils to participate in the pathogenesis of diabetes. Our previous study also found that the serum PR3 level of LADA patients was 4 times higher than that of healthy controls (81). Injection of recombinant PR3 can induce blood glucose increase in mice (82). A recent study found that the inhibition of NE secreted by neutrophils can reduce the infiltration of macrophages and reduce the autoimmune destruction of β cells mediated by cytotoxic T cells (83). Therefore, neutrophils may participate in the pathogenesis of LADA, but elucidating its specific mechanism requires further research.

##### 2.2.1.3 NK Cells

NK cells are innate immune cells with direct cytotoxicity against infectious pathogens (84, 85). They can also secrete different types of cytokines and regulate antigen presentation and T cell activation (84). Clinical studies have found that killer Ig-like receptors (KIRS) expressed on NK cells are related to the susceptibility and protection of adults in LADA patients in Latvia and Asia (86). Previous publications showed contradictory results for NK cell frequency in LADA patients compared with control groups. However, most studies have reported an increase in the frequency of circulating NK cells in LADA (17, 87, 88). This difference may be caused by the difference in the disease course of LADA patients in each study. In addition, the percentage of NKp46^+^ NK cells in peripheral blood of LADA patients was negatively correlated with fasting plasma C-peptide levels, which indicates that NKp46^+^ NK cells may play a role in the pathogenesis of LADA (17). The role of NK cells in autoimmune diabetes and its mechanism of action has not yet been elucidated. This may due to the researches focuse more on the total number of NK cells rather than subgroups. NK cell subgroups function diversely and can be used as targets for immunotherapy.

#### 2.2.2 Innate Immune Modulators and LADA

TLRs are the most characteristic membrane-bound PRRs, and they participate in the host defense against aggressive extracellular pathogens (89). To date, 10 human TLRs and 13 mouse TLRs have been identified. Among them, TLR1, TLR2, TLR4, TLR5, TLR6, and TLR10 are located on the cell surface. They can recognize pathogen-associated molecular patterns (PAMPs) such as triacylated lipoprotein, diacylated lipoprotein, lipopolysaccharide and flagellin, and damage-related molecular patterns (DAMPs) such as heat shock protein, HMGB1, and proteoglycan (90, 91). TLR3, TLR7, TLR8, and TLR9 are located in the endosome, which can recognize PAMPs such as viral single-stranded DNA, viral and bacterial double-stranded DNA, and DAMPs such as immune complex self-RNA and chromatin immune complex self-RNA (92). TLRs can be expressed by a variety of immune cells, such as neutrophils, macrophages, DCs, NK cells, T cells, and B cells, as well as various nonimmune cells including pancreatic β cells. Under infection, stress or injury conditions, TLRs recruit specific adaptors such as myeloid differentiation primary response protein 88 (MyD88), MYD88-adaptor-like (MAL, also known as TIR domain-containing adaptor protein or TIRAP), which contains the adaptor protein of the TIR domain induces interferon-β (TRIF), TRIF-related adaptor molecules (TRAMs) and SARM protein (sterile-α-and armadillo motif-containing protein) to induce downstream inflammation cascades and the production of type 1 interferon (93, 94). Under certain circumstances, inappropriate activation of TLRs by self-antigens contributes to chronic inflammation, as well as to systemic autoimmune diseases.

To date, studies targeting TLRs in autoimmune diabetes have focused on TLR2, TLR3, TLR4, TLR7 and TLR9. Among them, TLR2 and TLR4 are the most characteristic TLRs in LADA-related research. However, previous studies have shown conflicting results regarding the expression levels of TLR2 and TLR4 in patients with autoimmune diabetes. Some studies have shown that in circulating monocytes, both TLR2 and TLR4 in patients with T1D and LADA are unregulated (67, 95–99), while others have shown that TLR4 expression in T1D is downregulated (100, 101), and the expression level of TLR4 in LADA CD14+ cells is higher than that in T1D (102). High glucose upregulates mRNA and protein expressions of TLR2 and TLR4 human macrovascular aortic endothelial cells (HMAECs); inhibition of TLR2 and TLR4 signals can attenuate inflammation induced by high glucose (103). In addition, apoptotic β cells with secondary necrosis cause the inflammatory response of macrophages through the TLR2/MyD88/NF-κB signaling pathway (104). Late apoptotic β cell destruction can stimulate the initiation of diabetic T cells through TLR2-dependent antigen-presenting cell activation. This may be one of the initial events in autoimmune diabetes (104, 105). Also, TLR2 and TLR4 may be an important immunological link between gut microbes and the development of autoimmune diabetes (100). Further research is needed to determine the mechanisms by which of TLR2 and TLR4 exert effects on in LADA.

### 2.3 Gut-Associated Immunity and LADA

The intestinal microbiota is necessary for the normal development of the immune system after birth. The niche in mice with intestinal microbiota is saturated, so invading pathogens are more difficult to colonize (106). Many microorganisms and their metabolites have immunomodulatory effects and play a vital role in immune development and function. Polysaccharide (PSA) from *Bacteroides fragilis* can maintain the balance of Th1/Th2 and guide the development of lymphoid organs (107). *Bacteroides fragilis* can regulate the homeostasis of host iNKT cells through sphingolipids (108). Some of the metabolites have been shown to also engage in innate and adaptive immune regulation. Short-chain fatty acids (SCFAs) are produced by symbiotic microorganisms and can be recognized by G protein-coupled receptors (GPCR), and the most abundant SCFAs in the mammalian intestine are acetate, propionate and butyrate (109). SCFAs are active substrates of intestinal epithelial cells, which are part of the mucosal immune system. It has been shown that exposure of neutrophils and monocytes to SCFAs can lead to inactivation of NF-κB and suppression of pro-inflammatory cytokines (110). Currently, GPR43, GPR41, GPR109A and olfactory receptor 78 (Olfr78) have been identified as SCFA receptors, of which GPR109A can be expressed in certain DCs and macrophages to make them more efficient in inducing differentiation of Tregs and IL-10-producing T cells, while GPR43 can be expressed in neutrophils and acts as a neutrophil chemotactic receptor (111, 112). SCFAs can also modulate cytokine expression and T cell function by inhibiting the activity of histone deacetylases (HDAC) and supplying acetyl groups for acetyl-CoA (113). By combining the characteristics of intestinal microbiota and its metabolites with human immunity, the intestinal microbiota or intestinal metabolites may become new targets for the treatment of human diseases.

An increasing number of studies have reported that gut microbiota is related to the pathogenesis of autoimmune diabetes. Fecal microbiota transplantation (FMT) in patients with new-onset T1D can effectively maintain residual β cell function and is accompanied by alterations in plasma metabolites, intestinal gene expression, T cell autoimmunity, and fecal microbiota composition (114). A multi-omics study showed correlations between gut microbiota, fecal metabolites, serum metabolites, and clinical phenotypes (including islet autoantibodies, glucose metabolism, islet function, and inflammatory factors) in LADA patients, and the patients with LADA displayed distinct gut bacterial characteristics, such as a severe deficiency in SCFA-producing bacteria (e.g., *Faecalibacterium* spp., *Roseburia* spp., and *Blautia* spp.) (22). A previous study also showed that *Blautia* were positively correlated with glycosylated hemoglobin levels, the number of autoimmune diabetes antibodies and the titers of (IA-2A) (115). The use of symbiotic bacteria of single species with beneficial metabolic and immune effects has been initiated as a new direction for the prevention and treatment of T1D, but whether it is also applicable in LADA is inconclusive.

The intestinal barrier mainly includes the mechanical barrier of the intestine, such as the tight junction formed by intestinal epithelial cells (IECs) and goblet cells (116, 117) and innate lymphoid cells (ILCs) in the intestinal mucosal tissue (118), mast cells (119), monocytes (118), and so on. The normal intestinal barrier is the basis for the intestinal immune system and the intestinal symbiotic flora to maintain dynamic balance (120). When intestinal barrier function is impaired, it may cause the translocation of microorganisms, and initiate a pro-inflammatory immune response *via* immune cells and mediators (120). Previous studies have shown that patients with T1D have increased intestinal permeability (121, 122). It has been observed that gut bacteria can specifically move to the pancreatic lymph nodes, contributing to the pathogenesis of autoimmune diabetes (123, 124). It has been demonstrated that IECs can secrete a range of mediators to regulate innate and adaptive immune cell populations (125). Activation of intestinal innate and adaptive immunity has been found in the local environment of the duodenum of T1D patients, and the perturbed innate immune function of IECs may promote the expansion and polarization of autoreactive T cells (126). In addition, a study found that ILCs stimulated by intestinal microbes induce expression of β-defensin 14 (MBD14) in pancreatic endocrine cells (127). MBD14 can induce regulatory macrophages by stimulating TLR2, which in turn induces protective Tregs and maintains the immune tolerance of the pancreas (127). However, no relevant research on intestinal permeability in LADA patients has been found. Whether intestinal immunity exerts a role in the development of LADA is still a question worth exploring.

## 3 Immunotherapies for LADA

**Figure 1:**
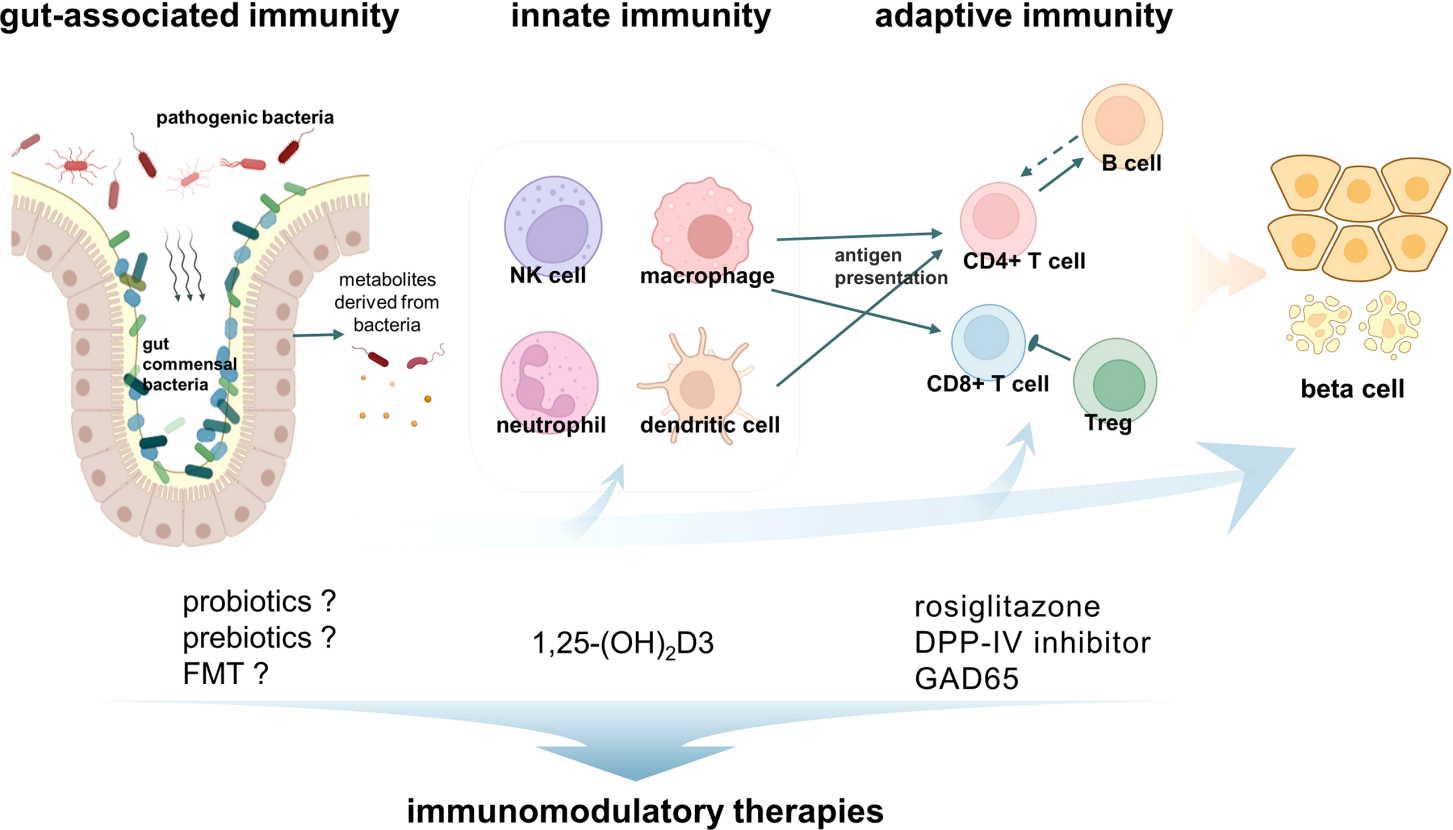
Immunity activates latent autoimmune diabetes in adults (LADA) and immunomodulatory therapies. The pathogenesis of LADA is the result of the interaction of innate immunity, adaptive immunity, and gut-associated immunity. Macrophages and CD8^+^ T cells are the most abundant immune cells infiltrating in the insulitis of LADA patients. In addition, other immune cells such as NK cells, neutrophils, CD4^+^ T cells, and B cells were also found to be involved in the development of LADA. A variety of corresponding immunomodulatory therapies have been developed.

Immunomodulatory therapy is ideal for LADA patients, who have a slower decline in pancreatic β cell function. Therefore, a series of immunomodulatory therapies targeting innate immunity, adaptive immunity, or gut microbiota have been conducted or are promising approaches in LADA (**Figure 1**).

### 3.1 Therapies Targeting Adaptive Immunity

Tripterygium polyglycoside is an immunosuppressant that has effects on both humoral and cellular immunity. Our previous study found that tripterygium polyglycoside has a regulatory effect on T cell subsets in LADA patients, which could inhibit the autoimmune response and improve pancreatic β cell function significantly (128, 129).

As an insulin sensitizer, there are a few clinical studies evaluating the safety and efficacy of rosiglitazone in LADA patients. Studies have found that rosiglitazone combined with insulin or not was beneficial for β cell function in patients with LADA (130, 131). Rosiglitazone has been shown to inhibit the inflammatory pathways mediated by nuclear factor of activated T cells (NF-AT) and NF-kB, and promote the regulatory potency of CD4^+^CD25^+^ T cells, thereby preventing immune destruction of β cells (131–133).

Dipeptidyl peptidase-IV (DPP-IV) is a serine exopeptidase. It is widely expressed on the surface of immune cells such as DCs, macrophages, T cells and activated B cells (134, 135). It is significantly upregulated upon T cell activation (134). Animal studies have shown that treatment with DPP-IV inhibitors can reverse new-onset diabetes in NOD mice by reducing insulitis, increasing CD4^+^CD25^+^FoxP3^+^ regulatory T cells, and stimulating β cell regeneration (136). Sitagliptin has been shown to increase the expression of IL-10 (an anti-inflammatory cytokine), and reduce the expression of pro-inflammatory cytokines, and cell adhesion molecules in patients with T2D (137, 138). Given the anti-inflammatory/immunomodulatory properties of DPP-IV inhibitors, it is possible that DPP-IV inhibitors exert multiple protective effects on pancreatic β cells. Patients with LADA exhibit higher DPP-IV activity compared with T1D and T2D, and DPP-IV activity is significantly associated with GADA titers in LADA (139). Previous studies have explored the role of DPP-IV inhibitors as an adjunctive therapeutic strategy to preserve β cell function in LADA patients. A double-blind, randomized, controlled study found that treatment with linagliptin slowed the rate of decline in C-peptide levels over a two-year disease trajectory in LADA patients, by increasing endogenous glucagon-like Peptide 1 (GLP-1) levels to protect β cells (140). Our randomized controlled studies showed that β cell function was preserved in LADA patients treated with sitagliptin plus insulin compared with insulin alone. Sitagliptin treatment altered the frequency of CD4^+^ T cell subsets reduced Th17 cells, elevated Th2 cells and downregulated the expression of pathological mRNAs, including RORC and T-BET (141, 142). In addition, we also found that saxagliptin was effective in lowering blood glucose levels and was well tolerated in GADA-positive patients (143, 144). Recent data suggested that supplement of 2000 IU/day of 1,25(OH)2D3 with saxagliptin could protect β cell function in LADA patients by inducing immune modulation (145). Larger randomized studies are necessary to demonstrate the role of DPP-IV inhibitors in pancreatic β cell protection in LADA patients.

Islet autoantibodies are a hallmark of autoimmune diabetes and are valuable tools to aid in the diagnosis of the disease. In a phase II clinical trial, two subcutaneous injections of recombinant human GAD65 formulated with aluminum hydroxide (GAD-alum) at 4-week intervals were safe in patients with LADA (146–148). Furthermore, therapy with GAD in diabetes has been shown to elicit durable immune responses (149). Compared with the placebo group, GAD65-induced expression of FOXP3 and TGF-β were increased in GAD-alum treated patients at 15 months, suggesting that Tregs may be responsible for the therapeutic effects (149). However, no significant effect has been observed in other clinical trials such as Diapep277 (150). In the future, antigen-specific treatment strategies should be individualized and more in line with precision medicine.

### 3.2 Therapies Targeting Innate Immunity

Vitamin D is a class of fat-soluble steroids, and its biologically active metabolite is 1 alpha, 25-dihydroxyvitamin D3 (1,25-(OH)_2_D3) (151). Vitamin D receptors are expressed on almost all immune cells, including neutrophils, T lymphocytes, and APCs such as DCs and macrophages (152). A growing number of studies have shown that 1,25-(OH)_2_D3 plays an important role in regulating innate and adaptive immune responses, leading to the activation of anti-inflammatory and immunomodulatory pathways and the induction of immune tolerance. A previous study by our group showed that 1,25-(OH)_2_D3 could regulate TLRs to downregulate NF-κB-p65 phosphorylation and significantly reduce IL-1β and TNF-α production (67). A Swedish-based case-control study found that taking vitamin D-rich fatty fish (≥1 time per week) may reduce the risk of LADA (153). Our prospective study demonstrated that 1-α(OH)D3 combined with insulin therapy protected pancreatic β cell function in LADA patient, and no serious side effects were observed in the 1-α(OH)D3 therapy or insulin plus 1-α(OH)D3 therapy over a follow-up period of more than 1 year (154). More prospective intervention studies are warranted to investigate the effectiveness of 1-α(OH)D3 as adjunctive therapy in the future.

### 3.3 Therapies Targeting Gut-Associated Immunity

Several environmental factors can trigger islet autoimmunity, leading to β cell apoptosis and possibly promoting the development of LADA (155). As an important environmental factor, the relationship between gut microbiota and LADA remains unclear. A recent cross-sectional study found significant differences in the gut microbiota between LADA patients and healthy subjects, patients with classic T1D and T2D (22). Moreover, there is a correlation between the gut microbiota and clinical phenotypes of LADA patients, suggesting that the gut microbiota is involved in the pathogenesis and progression of LADA (22). More research is needed to confirm whether interventions such as diet (probiotics, prebiotics dietary fiber supplements, etc.) and fecal transplantation can prevent the development of LADA by modulating the gut microbiota and affecting intestinal permeability.

## 4 Concluding Remarks

In recent years, the incidence and prevalence patients with adult-onset autoimmune diabetes have risen precipitously. LADA, which accounts for the majority of adult-onset autoimmune diabetes, has features of both T1D and T2D. Due to disease heterogeneity, it is difficult to determine the optimal treatment regimen for these diseases, and treatment should be individualized according to the characteristics of each LADA patient. The treatment of LADA mainly includes insulin therapy or a combination of insulin and other types of hypoglycemic drugs. Some patients with LADA experience a rapid decline in β cell function or are more likely to suffer from diabetic complications. The general goal of LADA treatment is metabolic control and preservation of residual insulin secretion function (3), and the development of new treatment methods is urgently needed.

In previous studies, great efforts have been made in animal experiments and clinical research to investigate the mechanism of innate and adaptive immunity in pancreatic β cell autoimmunity. In this review, we focus on the research progress related to innate immunity, adaptive immunity and intestinal microbiota in the context of LADA, and the current therapies targeting them. Therapeutic drugs for the immunity system of T1D have proven effective in animal experiments (96, 156, 157), and promising results have been obtained in clinical trials (158); however, the long-term side effects of the medications tested in these trials are unknown (159). The current data on LADA immune interventions are very limited and more extensive long-term and large-scale studies are needed. Previous studies on immunity focused on T1D, and mechanism studies were mainly done in animal models of T1D. The dearth of research on this topic may be due to the previous lack of an animal model for LADA; and the first animal model of LADA has been successfully constructed only recently (33). Therefore, the specific immunological mechanism in LADA may need to be verified in the animal model of LADA and then in patients, which will facilitate the design of immunity-related approaches to prevent and treat LADA.

## Author Contributions

JH wrote the manuscript. RZ, HZ, LX, and ZZ edited and revised the manuscript. YX revised the manuscript and provided critical feedback. All the authors approved the final version of the manuscript.

## Funding

This work was supported by the National Key Research and Development Program of China (2018YFE0114500 to YX), the National Natural Science Foundation of China (81820108007 to ZZ), the National Natural Science Foundation of China (81870577 to YX), the National Science Foundation of Hunan Province for Excellent Young Scholars (2020JJ3056 to YX), the science and technology innovation Program of Hunan Province (2021RC3032 to YX), the Natural Science Foundation of Hunan Province (2022JJ40689 to JH), and the Natural Science Foundation of Changsha (kq2202404 to JH).

## Conflict of Interest

The authors declare that the research was conducted in the absence of any commercial or financial relationships that could be construed as a potential conflict of interest.

## Publisher’s Note

All claims expressed in this article are solely those of the authors and do not necessarily represent those of their affiliated organizations, or those of the publisher, the editors and the reviewers. Any product that may be evaluated in this article, or claim that may be made by its manufacturer, is not guaranteed or endorsed by the publisher.
